# *N*-Acetyl-*d*-Glucosamine Kinase Interacts with NudC and Lis1 in Dynein Motor Complex and Promotes Cell Migration

**DOI:** 10.3390/ijms22010129

**Published:** 2020-12-24

**Authors:** Md. Ariful Islam, Ho Jin Choi, Raju Dash, Syeda Ridita Sharif, Diyah Fatimah Oktaviani, Dae-Hyun Seog, Il Soo Moon

**Affiliations:** 1 Department of Anatomy, Dongguk University College of Medicine, Gyeongju 38066, Korea; arif_du_ph@yahoo.com (M.A.I.); chjack@naver.com (H.J.C.); rajudash.bgctub@gmail.com (R.D.); ridita_sharif@yahoo.com (S.R.S.); diyahfatimah.oktav@gmail.com (D.F.O.); 2 Department of Biochemistry, Dementia and Neurodegenerative Disease Research Center, Inje University College of Medicine, Busan 47392, Korea; daehyun@inje.ac.kr; 3 Dongguk Medical Institute, Dongguk University College of Medicine, Gyeongju 38066, Korea

**Keywords:** dynein, Lis1, NAGK, neuronal migration, NudC

## Abstract

Recently, we showed that *N*-acetylglucosamine kinase (NAGK), an enzyme of amino sugar metabolism, interacts with dynein light chain roadblock type 1 (DYNLRB1) and promotes the functions of dynein motor. Here, we report that NAGK interacts with nuclear distribution protein C (NudC) and lissencephaly 1 (Lis1) in the dynein complex. Yeast two-hybrid assays, pull-down assays, immunocytochemistry, and proximity ligation assays revealed NAGK–NudC–Lis1–dynein complexes around nuclei, at the leading poles of migrating HEK293T cells, and at the tips of migratory processes of cultured rat neuroblast cells. The exogenous expression of red fluorescent protein (RFP)-tagged NAGK accelerated HEK293T cell migration during in vitro wound-healing assays and of neurons during in vitro neurosphere migration and in utero electroporation assays, whereas NAGK knockdown by short hairpin RNA (shRNA) delayed migration. Finally, a small NAGK peptide derived from the NudC interacting domain in in silico molecular docking analysis retarded the migrations of HEK293T and SH-SY5Y cells. These data indicate a functional interaction between NAGK and dynein–NudC–Lis1 complex at the nuclear envelope is required for the regulation of cell migration.

## 1. Introduction

Cell migration takes place during diverse developmental and pathogenic processes in multicellular organisms. Neuron migration during embryonic brain development essentially guides the complex layering of many structural and functional compartments. Neuronal progenitor cells (NPCs) are generated in spatially restricted ventricular zones (VZs) and undergo long-distance migration to reach their final destinations, e.g., the cortical plate (CP), to establish functional integrated neural circuitry [1,2]. NPCs migrate into the nearby subventricular zone (SVZ) and subsequently adopt a bipolar morphology [3]. Bipolar neurons extend a single or branched “migratory” leading process in the direction of movement. One or more transient “swellings” or “dilations” are formed within these leading processes that represent sites of attachment to underlying radial glial cells in vivo, and these dilations are also observed in migratory bipolar cells of dissociated neuronal cultures in vitro and in other cases of non-glial guided migration [4,5]. As leading processes advance, centrosomes move toward a swelling [6], and the cell body subsequently catches up with movement in a series of discontinuous steps, i.e., by nucleus–centrosome (N-C) coupling [4,5].

Dynein complexed with lissencephaly 1 (Lis1) and doublecortin (DCX) mediates N–C coupling during neuronal migration, and dynein heavy chain generates nuclear movement [7], while Lis1 potentiates dynein/dynactin functions [8]. It has been well established that dynein functions in retrograde cargo transportation and play essential roles in diverse signaling processes during embryonic development, which is predominantly influenced by the dynein association with Lis1 and NudE [9,10,11]. Mutations in NudE or Lis1 result in defects of brain development in mice [12,13,14]. Recent biochemical and biophysical studies indicate that the Lis1, NudE, and cytoplasmic dynein form a complex. Lis1, which is positioned adjacent to NudE in dynein, converts dynein to a persistent force-producing state, which is probably required to transport large structures, such as nuclei [15]. Nuclear distribution protein C (NudC) is a relatively uncharacterized Aspergillus gene in the cytoplasmic dynein pathway, although mammalian NudC is known to coimmunoprecipitate with dynein, dynactin, and Lis1 [16]. NudC, similar to Lis1, is required for neuronal migration during neocorticogenesis, and it has been reported NudC overexpression doubled the nuclear migration rate, and that NudC RNAi decreased movement in an in utero electroporation assay [17]. During nuclear migration, dynein and Lis1 may act from the nuclear surface. The nuclear envelope is decorated with dynein, Lis1, and their cofactors during nuclear envelope breakdown in mitotic prophase, which supports this suggestion [18,19,20]. Thus, studies suggest that NudC with cytoplasmic dynein participates in neuronal migration. 

Recently, we identified a novel dynein interacting protein N-acetylglucosamine (GlcNAc) kinase (NAGK; EC 2.7.1.59). This enzyme phosphorylates GlcNAc to produce GlcNAc-6-phosphate, which is a component that is utilized in uridine diphosphate N-acetylglucosamine (UDP–GlcNAc) biosynthesis or energy metabolism in the GlcNAc recycling/salvage pathway. Human NAGK monomer is 37 kDa sized, composed of N-terminal (small) and C-terminal (large) domains [21], belongs to the sugar kinase/heat shock protein 70/actin superfamily [22], and is characterized by a V-shaped fold consisting of two domains. Findings from our laboratory and other research group suggest a critical role of NAGK in the early development process [23,24,25]. NAGK is expressed at high levels in neurons but at low levels in astrocytes and oligodendrocytes [26,27], and it plays essential roles during the development of dendrites [26,27] and axons [25], in protein aggregates clearance [28], and during cell division [24]. NAGK interacted with dynein light chain roadblock 1 (DYNLRB1) during yeast two-hybrid selection [29]. Furthermore, immunostaining shows the two proteins colocalize in neuronal and non-neuronal cells in culture and brain tissue, and proximity ligation assays (PLAs) [29]. Interestingly, interruption of the NAGK–dynein interaction by the overexpression of NAGK small domain [26] or the introduction of a peptide derived from the C-terminal DYNLRB1 nullified the effects of NAGK on axonal [25] and dendritic outgrowths [29] as well as aggregates clearance [28]. Moreover, a kinase-deficient mutant NAGK also upregulated dendritic arborization [26], which confirmed that non-canonical, i.e., kinase-independent, and structural functions of NAGK promote multiple facets of dynein function.

During cell division, NAGK interacts with the perinuclear Dynein–Lis1–NudE1 complex during prophase nuclear invagination and with kinetochores during metaphase chromosome separation [24]. These findings suggest that NAGK plays an essential role in the molecular tug of war required for nuclear envelope breakdown and chromosome separation, during which the dynein motor complex is aided by bridging proteins such as NAGK, NudC, and Lis1 [30]. A common or similar molecular mechanism may underlie substrate attachment and force generation by dynein complex on microtubules (MT) during prophase nuclear invagination and subsequent nuclear envelope breakdown, chromosome separation, and cell migration. In this study, we investigated whether NAGK participates in cellular migration, which ostensibly is another type of intracellular tug-of-war, in cooperation with NudC and Lis1 on dynein complex.

## 2. Results

### 2.1. NAGK Interacted with NudC in the Yeast Two-Hybrid Screen and by Protein–Protein Docking Simulation

**Figure 1:**
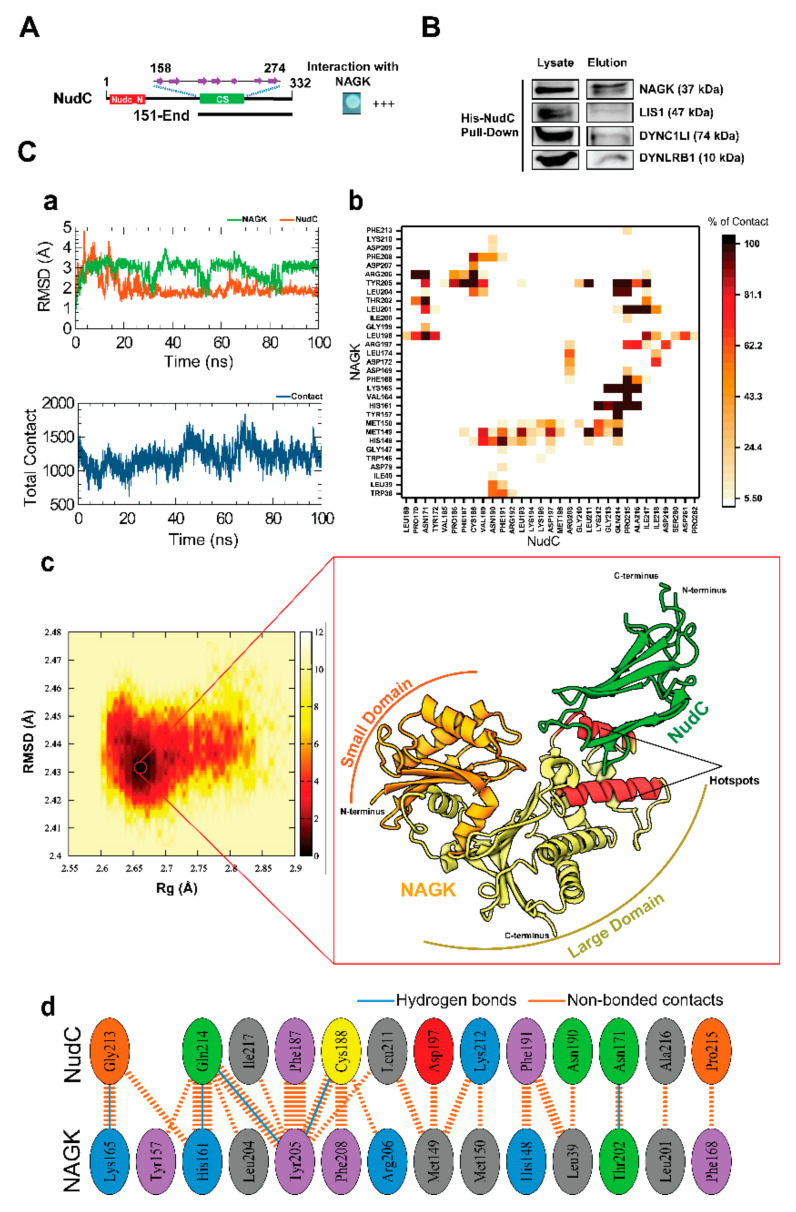
Verification of interaction of *N*-acetylglucosamine kinase (NAGK) with nuclear distribution protein C (NudC). (**A**). Yeast two-hybrid assay showing the domain of NudC interacting with NAGK. The NAGK small domain was used as bait. The C-terminal binding region (amino acids 151 to end) of NudC, which contains the CS-domain, is shown as a bar diagram. (**B**). Interactions of NudC with NAGK and dynein in HEK293T cells. HEK293T cells were transfected with His-tagged NudC plasmid and then cultured 48 h and lysed for nickel NTA (Ni-NTA) based pull-down assays. The interacted proteins were detected by immunoblotting with respective antibodies. (**C**). In silico protein–protein docking and molecular dynamics simulation revealed critical NAGK–NudC interaction hotspots. Here, the stability of the simulation system was confirmed by Root Mean Square Deviation (RMSD) analysis, which was performed by comparing the backbone atoms (C, Cα, and N) of individual proteins with their initial structure of the simulation (Upper Panel) (**Ca**). The total number of non-bonded contacts (lower panel) formed between NAGK–NudC during the 100 ns simulation. Percentage of intermolecular contacts summarized in the heatmap represents magnitudes of inter-residue contacts during the simulation. Here, the red to white color-coded bar represents higher to lower total contact percentages, respectively (**Cb**). Conformational dynamics results of NAGK–NudC complex are presented as a free energy landscape map, where dark red regions indicate the distribution of the conformer with the lowest energy minimum (0 kJ/mol). Subsequently, the most stable conformer of the complex derived from the free energy landscape, which was available at a corresponding timescale of 42.55 ns, and depicted in the Cartoon model (**Cc**), and their intermolecular interaction pattern is highlighted in the 2D plot, which describes major non-bonded interactions at the protein-protein interface (**Cd**).

The yeast two-hybrid selection assay showed that NAGK strongly interacted with the C-terminal of NudC (from aa151-End, Figure 1A). The C-terminal contains a CS-domain (PFAM accession number: PF04969) (named after CHORD-containing proteins and SGT1) [31]. This bipartite domain is of ≈100 residues, and its protein–protein interaction module is composed of a compact antiparallel β-sandwich fold consisting of seven β-strands. To confirm this interaction, a pulldown assay was conducted targeting the His-tagged NudC protein, which was performed by transiently transfected His-tagged NudC plasmid in HEK293T cells. The pull-down assay followed by immunoblotting with respective antibodies showed that exogenous NudC interacted with endogenous NAGK (Figure 1B). As both NAGK and NudC were reported to interact with dynein by previous studies [16,28], we also checked the presence of dynein components. Interestingly, both DYNLRB1 and light intermediate chain 1 (DYNC1L1) were found to interact with exogenous NudC. Moreover, immunoblot also showed the presence of Lis1 in the pull-down elution, which confirms that NAGK interacts with NudC and Lis1 in the dynein complex.

To achieve insight of the interaction between NAGK and NudC, we considered protein–protein docking simulation, which predicts complex structures from individual proteins. Accordingly, a full blind docking simulation was carried out to detect a putative binding site for NAGK in NudC. The docked complex obtained revealed a binding energy of −8.79 kcal/mol with a docking score of −29.35 kcal/mol (Figure S1A). The complex was further subjected to an additional 100 ns molecular dynamics simulation. Root Mean Square Deviation (RMSD) analysis was performed on the simulation trajectories, and both proteins in the complex were found to achieve equilibration after 20 ns and then remained stable until the end of the simulation (Figure 1Ca). However, the backbone RMSD values of NudC within the complex exhibited large fluctuations, which were attributed to free movement of the C-terminal domain, as revealed by the root mean square fluctuation (RMSF) study (Figure S1B). Overall, RMSD analysis indicated that resultant trajectories provided an appropriate basis for further analysis. Hence, we calculated total contact between NudC and NAGK formed during the 100 ns simulation. As shown in Figure 1Ca, a considerable number of interactions were observed between NAGK and NudC. Numbers of contacts increased after 40 ns, and the fluctuation level was maintained until the end of simulation. For more insight, an additional heat map was produced based on percentages of total contacts and highlighting hotspots in the protein–protein interaction interface (Figure 1Cb). The map showed that several residues in the two major regions of the large domain of NAGK (residues from Lys^198^ to Phe^208^ and Tyr^157^ to Ile^171^) interacted potently with the NudC CS domain. To improve understanding, free energy landscape analysis was conducted on the resultant trajectories to identify the most stable complex structure with the lowest energy minimum (Figure 1Cc). Then, this structure was subjected to detailed intermolecular interaction analysis, which showed that residues Thr^202^, Leu^204^, Tyr^205^, and Phe^208^ in the Lys^198^ to Phe^208^ region of NAGK maintained hydrogen bond interactions and non-bonded contacts with NudC (Figure 1Cd). As regards the Tyr^157^ to Ile^171^ region, Lys^165^ and His^161^ also maintained hydrogen bond interactions and non-bonded contacts with NudC. Moreover, both of these major regions of NAGK (Tyr^157^ to Ile^171^ and Lys^198^ to Phe^208^) maintained strong interactions with NudC during simulation, especially the Leu^211^ to Ile^218^ region, which indicated the presence of a major protein-binding interface in NudC. 

### 2.2. NAGK Colocalized with NudC during Migration

**Figure 2:**
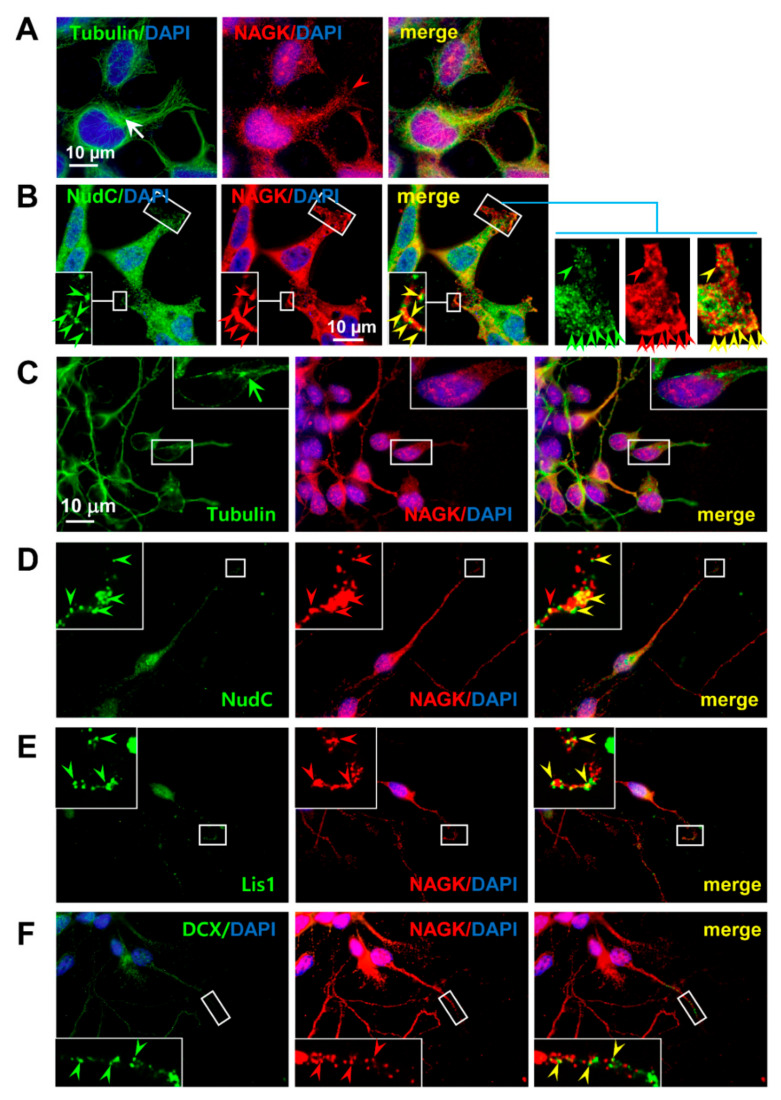
Colocalization of NAGK with NudC and lissencephaly 1 (Lis1). (**A**). HEK293T cells grown on coverslips were subjected to immunofluorescence analyses with the indicated antibodies. DNA was visualized by 4′,6-diamidino-2-phenylindole (DAPI) staining, and centrosomes by staining with anti-tubulin antibody (arrow). (**B**). Boxed areas are enlarged (*insets*) on the right to show colocalization between NAGK and NudC signals (arrowheads) at the leading protrusions of migratory cells. (**C**). Double labeling for NAGK and tubulin: Boxed areas are enlarged (*inset*) to show the stronger tubulin signal at the centrosome (green arrow) in front of the nucleus with respect to migration direction. NAGK was localized to dendrites and in nuclei of migrating neurons. (**D**). Double labeling for NAGK and NudC: An enlarged (*inset*) representative leading projection of a migrating neuron showing the colocalization of NAGK signals (red arrowhead) and NudC immunosignals (green arrowhead). (**E**). Double labeling for NAGK and Lis1: Colocalizations of NAGK with Lis1 signals observed in leading neurites of a migratory neuron are indicated by yellow arrowheads (*inset*). (**F**). Double labeling for NAGK and doublecortin (DCX): Colocalization of NAGK and DCX signals in the leading dendritic projections of the migratory neuron are shown by yellow arrowheads (*inset*). Scale bar; 10 μm.

NudC has a well-established role in migration, and thus, we examined the in situ colocalization of NAGK and NudC in migrating cells. First, semi-confluent (more than 95%) HEK293T cells were scratched as mentioned in Material and Methods, and then, cells were allowed to migrate toward “wounds” on coverslips. Then, immunocytochemistry (ICC) was performed using anti-NAGK and anti-β-tubulin primary antibodies to determine the migratory morphology of cells and the distribution of NAGK. Intracellularly, centrosomes were located in front of the nuclei with respect to the migration direction (Figure 2A, white arrow), and MTs formed cage-like perinuclear structures. In leading processes, NAGK distribution was similar to those of MTs (Figure 2A, red arrowhead). At this migratory stage, NudC expression was highest around nuclei and gradually reduced to cell peripheries (Figure 2B). At the leading edges of migratory protrusions, cluster-like NudC signals were observed colocalized with NAGK immunoreactive (IR) puncta (Figure 2B, *inset*, right panel, arrowheads). These findings indicate NAGK–NudC interacted at the leading edges of cell cortices during migration. 

Our laboratory previously reported NAGK interacted with DYNLRB1 at neuronal dendrites [29], whereas others have reported NudC interacted with Lis1 and the dynein motor on the leading projections of migrating neurons [32]. Therefore, we examined the colocalizations of NAGK with NudC and Lis1 in migratory neurons. To do this, we prepared neurons migrating out of neurospheres generated from E14 neuronal progenitor cells (NPCs). First, we performed NAGK–tubulin double staining and found that centrosomes, identified by strong tubulin signals, were located rostral to nuclei with respect to migration direction (Figure 2C, green arrow). NAGK–IR signals were also observed in dendrites, on nuclear membranes, and within nuclei, as our laboratory previously showed [27,33]. When these migrating neurons were double-stained with anti-NAGK and anti-NudC or anti-Lis1 antibodies, NAGK IR signals were colocalized with NudC (Figure 2D, inset, arrowheads) and Lis1 signals (Figure 2E, inset, arrowheads) at leading neurite tips. When neurons were immunostained with anti-doublecortin (DCX) antibody (a marker of migratory neurons) to confirm the migratory phase, colocalization of NAGK and DCX were observed (Figure 2F, inset, arrowheads). These findings indicate NAGK, NudC, Lis1, and DCX interact during neuronal migration.

### 2.3. Endogenous NAGK–NudC Complex Colocalized with DYNLRB1 during Migration

**Figure 3:**
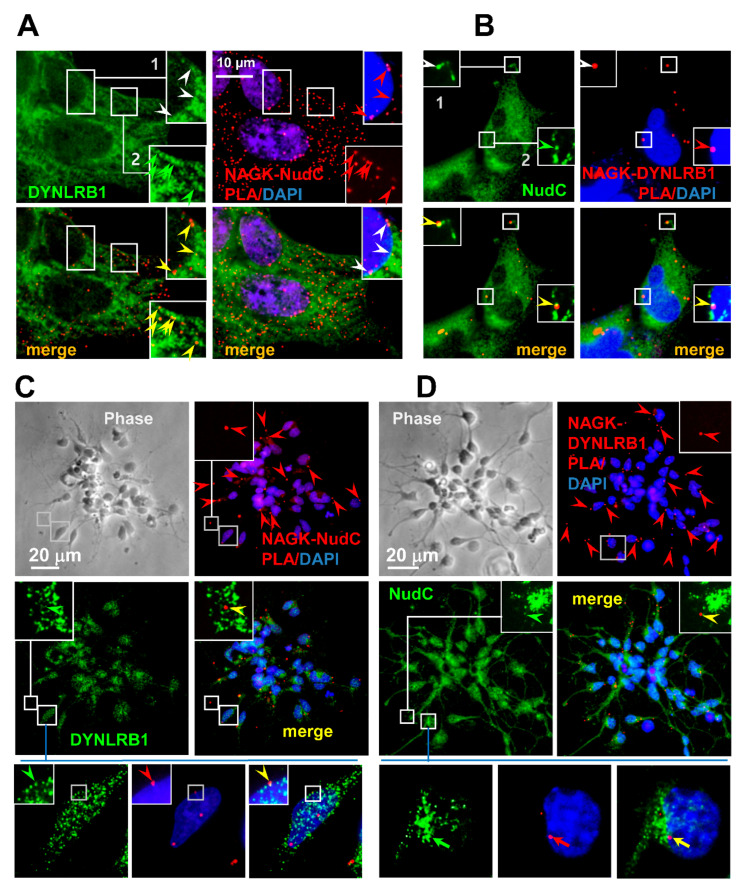
Co-localizations of NAGK, NudC, and dynein light chain roadblock type 1 (DYNLRB1) at nuclear membranes and on cell cortices. Proximity ligation assays (PLAs) were performed on migratory HEK293T cells (A, B) or migratory neurons (C, D, E, F). (**A**). NAGK–NudC PLA was performed and followed by immunostaining (ICC) with anti-DYNLRB1 antibody (green). Nuclei were visualized by DAPI staining (blue). The positions of PLA signals at a nuclear membrane (inset 1) and on a cell cortex (inset 2) are marked with boxes and enlarged to show the colocalization of NAGK–NudC PLA signals (red arrowheads) and DYNLRB1 ICC puncta (green arrowheads). Scale bar: 10 µm. (**B**). NAGK–DYNLRB1 PLA signals (red) colocalized with NudC ICC signals (green) in cell cortices (inset 1 of the merge, yellow arrowhead) and at nuclear membranes (inset 2 of the merge, yellow arrowhead). Scale bar: 10 µm. (**C**). In migrating neurons, the NAGK–NudC PLA signal (red arrow) colocalized with DYNLRB1 ICC puncta (green arrow) in the leading projection (enlarged in insets) and around the nucleus (lower panels). Scale bar; 20 µm. (**D**). Colocalizations of NAGK–DYNLRB1 PLA dots (red arrow) with NudC ICC signals (green arrow) at leading neuritic projections (inset) and on the nuclear membrane (arrow in lower panels) are shown. Scale bar; 20 µm.

To investigated endogenous NAGK–NudC interactions in migrating HEK293T cells, we performed in situ PLAs (proximity-ligation assays) using NAGK and NudC primary antibodies. To visualize NAGK–NudC–DYNLRB1 interactions, PLA was followed by ICC using anti-DYNLRB1 antibody, and nuclei were counter-stained with DAPI. NAGK–NudC interactions (red signals) due to PLA reactions were observed around nuclei (Figure 3A, *inset* 1, red arrowheads) and at cell cortices (*inset* 2, red arrowheads), and these were found to colocalize with DYNLRB1 ICC signals (green arrowheads). These results show NAGK interacted with NudC and dynein around nuclei and at cell cortices during cell migration. Next, NAGK–DYNLRB1 PLA was performed, followed by NudC ICC and DAPI counter-staining (Figure 3B). PLA signals for NAGK–DYNLRB1 complexes (red arrowheads) were colocalized with NudC ICC signals (green arrowheads) on leading migratory projections of cell cortices (Figure 3B, inset 1, arrowheads) and around the nuclei of migrating cells (Figure 3B, inset 2, arrowheads). These results confirm that a tripartite NAGK–NudC–DYNLRB1 interaction occurs during migration and suggest that NAGK might regulate functions of the dynein–NudC interaction during nuclear movement (i.e., N–C coupling) and cell migration.

Next, we examined the NAGK–NudC interaction in cultured neurons migrating from neurosphere aggregates. PLAs were conducted on NAGK–NudC or NAGK–DYNLRB1 complexes, which were followed by ICC for the third protein of this tripartite complex. NAGK–NudC PLA interactions (red dots) were colocalized with DYNLRB1 ICC signals (green) in leading dendritic projections (Figure 3C, *inset*, arrowheads) and near nuclear membranes (lower panel, arrowheads), which suggested that NAGK participated in the neuronal migration in association with the NudC–Dynein complex. NAGK–DYNLRB1 PLA signals (red) also colocalized with NudC ICC signals (green) in leading neurites (Figure 3D, inset, arrowheads) and around nuclear envelopes (lower panel, arrows). NAGK–DYNLRB1 PLA signals showed a distribution pattern similar to that previously reported [25,29]. These findings indicate that NAGK interacts with dynein–NudC complex during neuronal migration and suggest that NAGK plays a regulatory role during this process.

**Figure 4:**
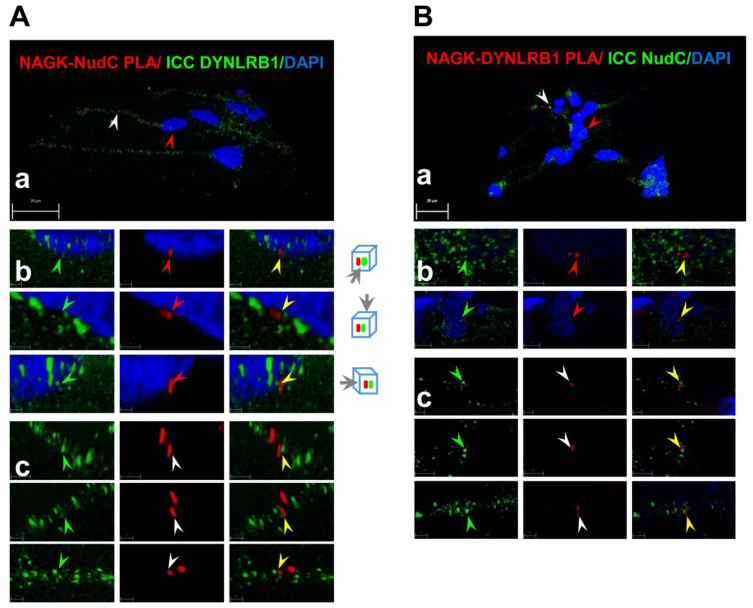
Co-localizations of NAGK, NudC, and DYNLRB1 at nuclear membranes and on cell cortices at high-resolution confocal 3D images. The colocalization of the NAGK–NudC complex (PLA signals) with DYNLRB1 ICC signals (A) and of NAGK–DYNLRB1 complex (PLA signals) with NudC ICC signals (N). Super-sensitive high-resolution confocal laser scanning microscopy (Leica TCS SP8) was used to capture *z*-stack images of the same slides shown in Figure 3C,D. Images were processed using the default 3D deconvolution algorithm built into the LAS X software to develop 3D models (**Aa**,**Ba**). Images captured from different angles (shown on the right of panel A) showing the colocalization of PLA and ICC signals on nuclear membranes (**Ab**,**Bb**) and in dendrites (**Ac**,**Bc**).

To confirm the hypothesis that NAGK forms a complex with NudC and dynein motor in migrating neurons, we examined the slide shown in Figure 3C,D under a super-sensitive high-resolution confocal laser scanning microscope (Leica TCS SP8). Images were processed using the default three-dimensional deconvolution algorithm in LAS X software. First, we took several *z*-stack images and developed a 3D model (Figure 4Aa, Ba). Then, we targeted one PLA signal around the nucleus (indicated by a red arrowhead for NAGK–NudC in Figure 4Ab and for NAGK–DYNLRB1 PLA in Figure 4Bb) and one dendrite (white arrowheads in Figure 4Ac,Bc). We found that PLA signals colocalized with corresponding ICC signals (Figure 4A, DYNLRB1, green arrowhead; Figure 4B, NudC, green arrowheads) when the signals were imaged at two or three different angles (representative directions are shown on the right of Figure 4Ab).

### 2.4. NAGK Interacted with NudC–Lis1 Complex during Migration 

**Figure 5:**
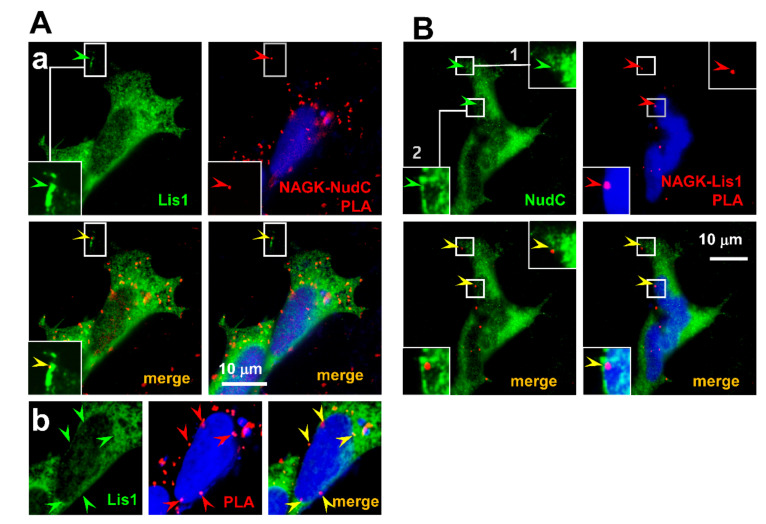

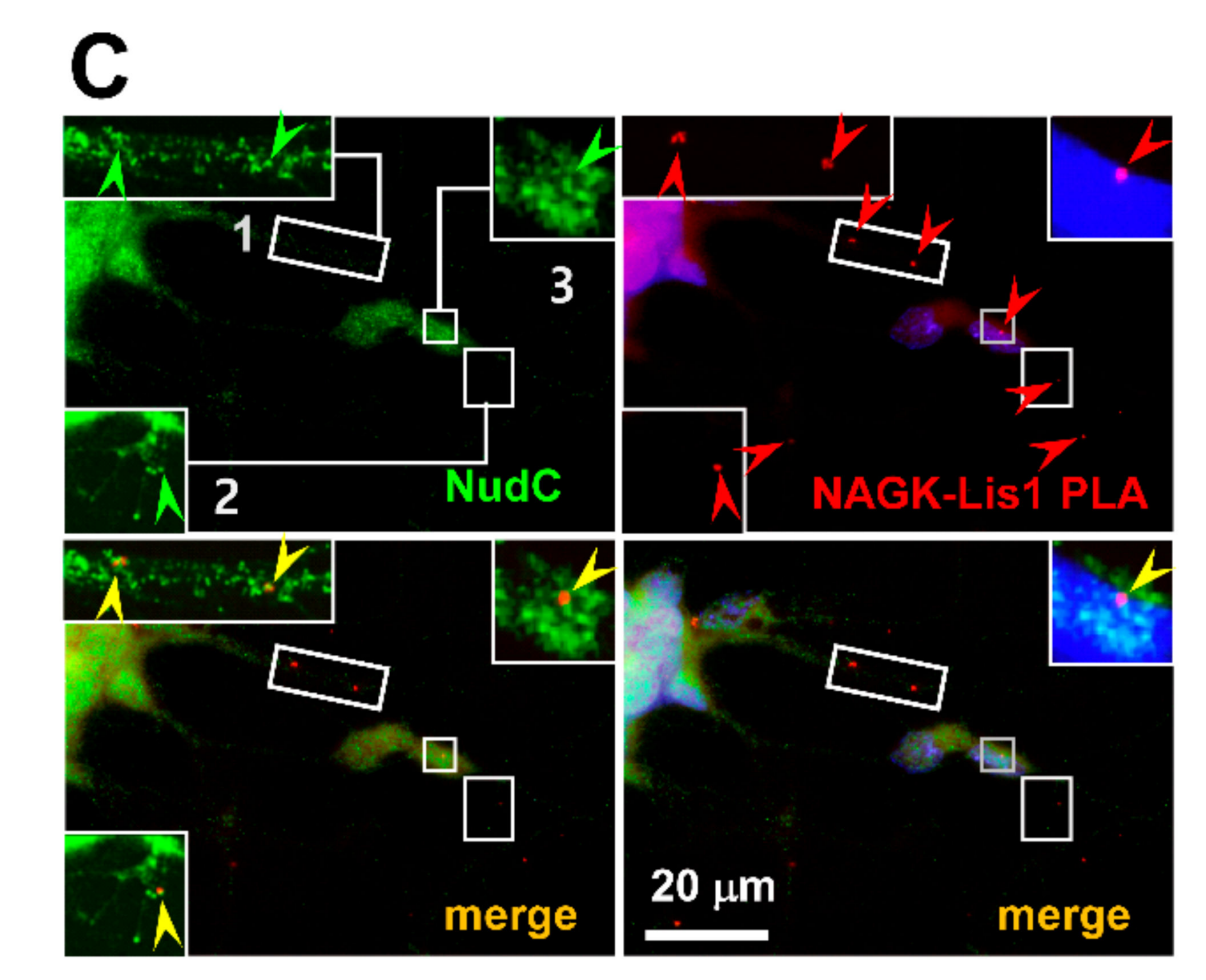
The NAGK–NudC–Lis1 interaction on nuclear membranes and cell cortices. (**A**). NAGK–NudC PLA was performed on migratory HEK293T cells and ICC with an anti-Lis1 antibody. Colocalization of PLA signals for NAGK–NudC complexes (red) with Lis1 ICC signals (green) in cell cortex (**Aa**, *inset*) and on the nuclear membrane (**Ab**, lower panel) are shown by arrowheads. (**B**). NAGK–Lis1 PLA followed by NudC ICC and DAPI staining was performed on migratory HEK293T cells. Representative NAGK–Lis1 PLA/NudC ICC and merged images are shown. Part of a cell cortex (box 1) and the small portion around a nuclear membrane (box 2) are enlarged (*insets*) to show the colocalization of the NAGK–Lis1 complex (red arrowheads) with NudC ICC signals (green arrowheads). Scale bar: 10 µm. (**C**). A set of experiments similar to those in 4B above was carried out on migrating neurons. Enlarged boxed areas show the colocalization of NAGK–Lis1 PLA (red arrowheads) with the immunosignal of NudC (green arrowheads) in dendritic projections (*insets* 1,2) and on the nuclear membrane (*inset* 3).

As NudC has been reported to interact with Lis1 during cell migration [34], we investigated the interaction between NAGK and the NudC–Lis1 complex in migrating HEK293T cells. PLA was conducted using anti-NAGK and anti-NudC primary antibodies followed by ICC with anti-Lis1 antibody in HEK293T cells during the migrating phase (Figure 5A). PLA signals (red arrowheads), representing NAGK–NudC interactions, colocalized with Lis1 ICC signals (Figure 5A, green arrowheads) in peripheral cell cortices (Figure 5Aa, inset, arrowheads) and around nuclei (DAPI counter-stained). To better show the relative positions of PLA signals (arrowheads) and nuclear boundaries, a *z* layer different from that of Figure 5(Aa) was shown in Figure 5Ab. To further confirm NAGK–NudC–Lis1 tripartite interactions, we conducted NAGK–Lis1 PLA followed by ICC for NudC with DAPI counter-staining (Figure 5B). NAGK–Lis1 interactions (PLA signals, red arrowheads) were observed to colocalize with NudC ICC signals (green) in peripheral cell cortices (*inset 1*, arrowheads) and around nuclei (*inset* 2, arrowheads). NAGK–Lis1 interactions were also observed in dendritic projections (Figure 5C, *inset* 1, 2, arrowheads) and at nuclear peripheries (Figure 5C, *inset* 3, arrowheads) colocalized with NudC signals in migrating neurons (Figure 5C, *inset*, arrowheads). Together, these results show the colocalization of NAGK–Lis1–NudC at nuclear peripheries, in cell cortices, and in leading projections of migrating neurons.

### 2.5. Exogenous NAGK Overexpression Accelerated but NAGK Knockdown Delayed Migratory Phenotype

**Figure 6:**
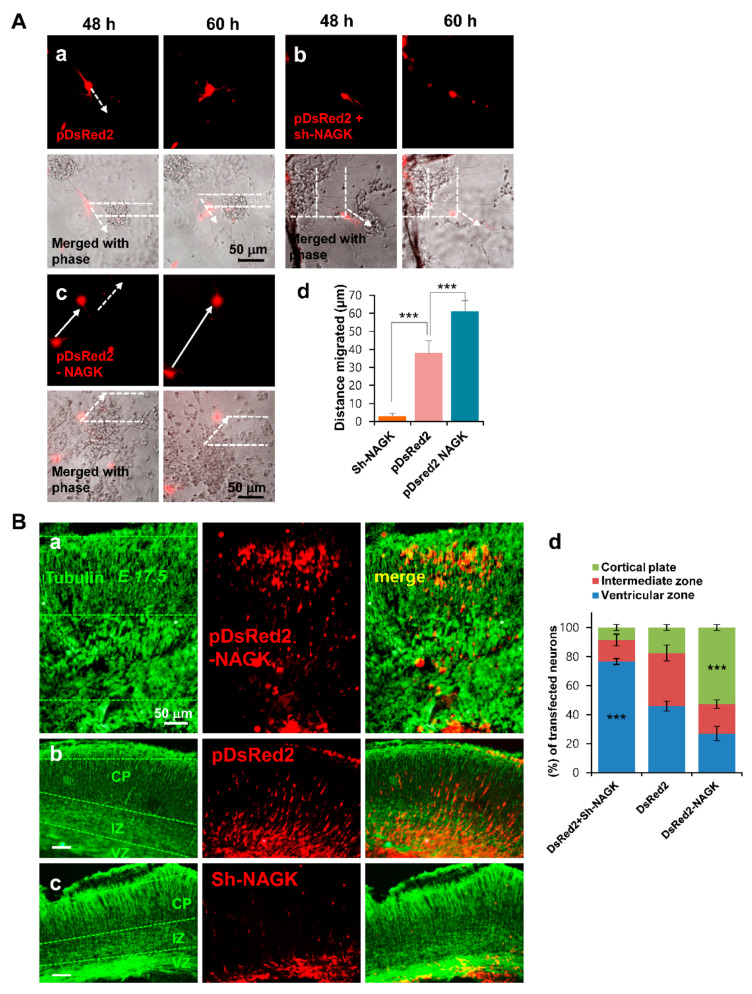
NAGK expression regulates neuronal migration. (**A**). Neurosphere migration assay. Neuronal progenitor cell (NPC) aggregates were transfected at time of plating with the indicated plasmids, and migration assays were performed as described in Materials and Methods. The white dotted line outlines the migratory movement of transfected neurons, and arrows show the direction of migration. Relative positions of migrating neurons transfected with pDsRed2 vector plasmid over a 12 h period (from 48 to 60 h) are shown by arrows (**Aa**). Neurons co-transfected with pDsRed2 vector and NAGK short hairpin RNA (shRNA) (sh-NAGK) migrated only slightly from aggregates and are indicated by the dotted lines (**Ab**). Neurons transfected with pDsRed2–NAGK migrated longer distances (**Ac**). The double-headed arrows show the movements of two transfected neurons. Scale bar; 50 µm. Distances migrated by transfected neurons from aggregates plotted as a bar diagram (**Ad**). The average distance traveled by neurons transfected with sh-NAGK was significantly less than that of neurons overexpressing NAGK, which in turn was significantly greater than that of neurons transfected with pDsRed2 (controls). *** *p* < 0.01, *n* = 5. (**B**). In utero electroporation of NAGK promoted neuronal migration in embryonic mouse brains. Neurons were transfected by in utero electroporation with either pDsRed2-NAGK (Ba), pDsRed2 vector, (**Bb**) or pDsRed2 plus NAGK sh-RNA (sh-NAGK) at embryonic day 14.5 (E14.5). Cryosections were imaged at E17.5. Tubulin immunostaining (green) showing the total distribution and morphology of neurons. Cortical plate (CP), intermediate zone (IZ), and ventricular zone (VZ) are marked with green dotted lines. Many neurons overexpressing DsRed2-NAGK moved to CPs (**Ba**), but NAGK knockdown neurons failed to move and mostly located at VZs (**Bc**). Neurons transfected with control plasmid moved toward CPs but most remained in VZs and in IZs (**Bb**). Transfected neurons in CP, IZ, and VZ zones were counted and percentages of total neurons were calculated (**Bd**). The percentages of neurons overexpressing NAGK were significantly higher in CPs than control neurons. In contrast, a significantly lower percentage of sh-NAGK shRNA transfected neurons were present in CPs than control neurons. *** *p* < 0.01, *n* = 3.

Neuronal migration has been reported to be compromised by dynein [6,35] or NudC [17] knock-out; therefore, we hypothesized that NAGK might also be involved in the neuronal migration. To investigate this possibility, we used in vitro migration assays using neurospheres generated from E14.5 rat embryonic cortices. NPCs were isolated from developing cortices (E14.5) and cultured as non-adherent neurospheres. To model neuronal migration, neurospheres were adhered to Matrigel coated with poly-L-lysine, and migration distances of transfected neurons from neurosphere boundaries were measured. Briefly, NPC aggregates (neurospheres in differentiation media) were plated and transfected with control pDsRed2 (Figure 6Aa), co-transfected with NAGK shRNA and pDsRed2 (Figure 6Ab), or transfected with pDsRed2-NAGK (Figure 6Ac). At 48 to 60 h after transfection, NAGK knockdown neurons migrated only slightly (Figure 6Ab). In contrast, neurons overexpressing NAGK migrated further (Figure 6Ac) than control neurons transfected with pDsRed2 (Figure 6Aa). We measured distances migrated by transfected neurons in three different experiments and found the average distance traveled by neurons transfected with shNAGK (≈58 µm) was significantly shorter than that of neurons transfected with pDsRed2–NAGK (≈61 µm), whereas neurons transfected with pDsRed2 control vector migrated ≈37 µm. This result indicates that NAGK has a regulatory role during neuronal migration.

Next, we used in utero electroporation to study the role of NAGK in neuronal migration in vivo during embryonic brain development. We introduced pDsRed2-NAGK (Figure 6Ba), pDsRed2 (Figure 6Bb), or NAGK shRNA plasmid and pDsRed2 together (Figure 6Bc) into the ventricular zone (VZ) of E14.5 mouse embryonic brains. The depletion of NAGK by shRNA retarded the movement of neurons from VZ to the cortical plate (CP) and transfected neurons localized predominantly to the VZ (indicated by red in Figure 6Bc), whereas the introduction of DsRed2–NAGK promoted migration and increased the numbers of transfected neurons in CPs (Figure 6Ba) as compared with pDsRed2 controls (Figure 6Bb). Transfected neurons in CPs, intermediate zones (IZs), and VZs were counted and plotted on a bar diagram (Figure 6Bd). The number of neurons in CPs was significantly increased by exogenous DsRed2-NAGK (by ≈50%) as compared with the DsRed2 control (≈18%). Furthermore, the percentage of migrating cells transfected with NAGK shRNA plasmid was significantly lower (≈5%). These results suggest that NAGK is essential for neuronal migration and embryonic brain development. 

It was reported that NudC depletion significantly inhibited collective cell migration in a wound-healing experiment [36]. To investigate the role of NAGK during migration, HEK293T cells were transfected with pDsRed2 control plasmid (Figure S2Aa, pDsRed2), a red fluorescent protein (RFP)-tagged NAGK plasmid (Figure S2Ab, pDsRed2-NAGK), or transfected with a pDsRed2 and NAGK shRNA plasmid (Figure S2Ac, sh-NAGK). Then, the movement patterns and shapes of transfected cells were observed, and images were captured. Cells that on migration front and following non-migrating cells were counted; examples are indicated as arrowheads and asterisks, respectively, in Figure S2Ab. Percentages of migratory cells were plotted as a bar diagram (Figure S2Ad). Most of the cells transfected with pDsRed2-NAGK plasmid were at the migration front. The majority (≈70%) of the transfected cells expressing exogenous NAGK migrated, and this was significantly greater than the percentage (≈40%) of pDsRed2 expressing controls that migrated. Furthermore, the percentage (≈14%) of migratory cells transfected with NAGK shRNA plasmid was significantly less. These results suggest that NAGK is essential for cell migration. 

### 2.6. A Peptide Derived from the In Silico NAGK–NudC Interacting Domain Retarded Neuron Migration

**Figure 7:**
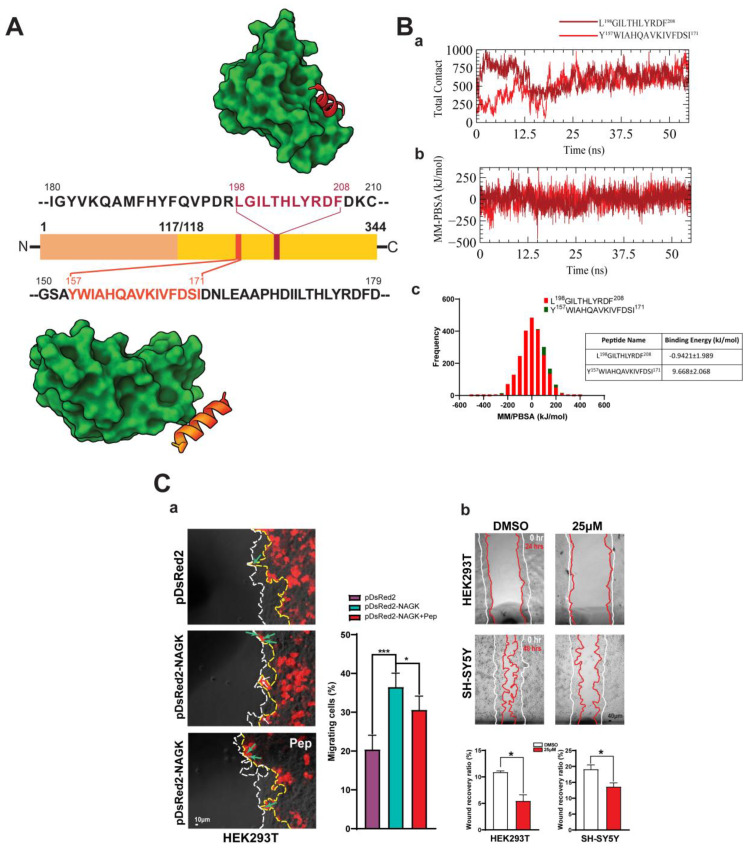
NudC to NAGK peptide binding and its effect on cell migration. (**A**). Mapping of peptide regions from the large domain of NAGK and their interactions with NudC. Based on molecular dynamics simulation analysis, two major hotspot regions were identified in NAGK, i.e., Y^157^WIAHQAVKIVFDSI^171^ (lower) and L^198^GILTHLYRDF^208^ (upper). Their interactions with NudC are represented by the 3D cartoon model. (**B**). Comparative binding energies of selected two peptides by 55 ns molecular dynamics simulation employing total contact analysis (**Ba**) and molecular mechanics/Poisson–Boltzmann Surface Area (MM-PBSA) binding energy calculations (**Bb**,**Bc**). Here in section (**Ba**,**Bb**), the red line indicates Y^157^WIAHQAVKIVFDSI^171^ and the brick red color L^198^GILTHLYRDF^208^. (**Bc**) The average binding energy of the NAGK derived peptide to NudC calculated considering all trajectories in 55 ns of molecular dynamics simulation. (**C**). Wound-healing assays. (**Ca**) Effects on NAGK-transfected cells. HEK293T cells were transfected with pDsRed2 (upper) or pDsRed2–NAGK (middle), and the latter was further transfected with 1 µg of Y^157^WIAHQAVKIVFDSI^171^ peptide (lower, Pep). Epifluorescent live-cell images showing transfected cells merged with phase-contrast cells. The white dotted line shows the migratory front, and the yellow dotted line outlines the migratory front of transfected cells. Scale bar; 10 µm. Bar diagram showing the percentage of migrating transfected cells at the leading edge. (**Cb**) Effects on migration when the peptide was added directly to the medium. HEK293T and SH-SY5Y cells were serum-starved for 4 h, and then, the medium was replaced with low (1.0%) serum medium containing DMSO or peptide (Y^157^WIAHQAVKIVFDSI^171^, 25 µM), and further incubated for the indicated times. Initial start positions (0 h) and migration fronts are marked by dotted white and yellow lines, respectively. The bar diagrams show wound-healing ratios (defined as the percentage of area covered by migrating cells divided by total wound areas). Scale bar; 40 µm. *** *p* < 0.0001, * *p* < 0.05, *n* = 5.

Since in silico protein–protein docking analysis indicated that NudC interacts with two regions of the large domain of NAGK, Y^157^WIAHQAVKIVFDSI^171^, and L^198^GILTHLYRDF^208^, we initially considered both regions for peptide design in order to confirm possible interactions with NudC binding domain (Figure 7A). Therefore, an additional 55 ns molecular dynamics simulation was performed for each peptide–NudC complex to determine binding feasibilities and binding energies (Figure 7Bc). For these simulations, a peptide–protein complex was designed from the lowest energy structure obtained by free energy landscape analysis and performed molecular dynamics simulation by following the similar method described previously for NAGK–NudC complex simulation. We analyzed the total contacts and binding energies of the peptide–protein complexes. Total contact analysis showed that both peptides maintained a similar number of interactions during the simulations (Figure 7Ba). However, molecular mechanics/Poisson–Boltzmann Surface Area (MM/PBSA) binding energy analysis showed that Y^157^WIAHQAVKIVFDSI^171^ had a higher affinity for NudC than L^198^GILTHLYRDF^208^ (binding energies were −0.942 kJ/mol and 9.668 kJ/mol, respectively) (Figure 7Bb,c, Lower panel). Hence, the peptide Y^157^WIAHQAVKIVFDSI^171^ was subsequently studied to determine whether its targeted binding site to NudC would influence the NAGK–NudC interaction and affect migration. 

To examine the effects of the selected peptide (Y^157^WIAHQAVKIVFDSI^171^) on cell migration, we performed two different types of wound-healing assays. First, we performed wound-healing assays after transfecting HEK293T cells with pDsRed2 or pDsRed2-NAGK and then further transfecting cells with the peptide. Percentages of migrating cells were calculated (defined as the percentages of transfected cells that migrated at migration fronts) after 48 h of incubation (Figure 7Ca). In the absence of peptide, ≈40% of pDsRed2-NAGK transfected cells migrated at the migration front, which was significantly (*p* < 0.0001) greater than that of DsRed2 transfected cells. Transfection with peptide Y^157^WIAHQAVKIVFDSI^171^ significantly reduced the portion of percentage of pDsRed2–NAGK transfected cells (pDsRed2-NAGK + pep), but this was still higher than that of pDsRed2-transfected cells, which implied incomplete inhibition. Next, we performed similar assays by adding Y^157^WIAHQAVKIVFDSI^171^ peptide (25 µM) directly to the culture medium. As shown in Figure 7Cb, treatment for 24 h significantly inhibited HEK293T cell migration, and treatment for 48 h significantly inhibited SH-SY5Y migration. These observations indicated that Y^157^WIAHQAVKIVFDSI^171^ specifically inhibited the NAGK–NudC interaction, thus affecting migration. Based on considerations of all observations, we propose that NAGK interacts with NudC and Lis1 on the dynein complex and promotes neuronal and non-neuronal cell migration.

## 3. Discussion

The present study shows that NAGK interacts with the NudC–Lis1–dynein complex and regulates cell migration. The first evidence of the involvement of NAGK in cell migration was obtained by a yeast two-hybrid screening and pull-down assay, which showed that NAGK interacted strongly with the C-terminal of mouse NudC (GI: 18221; a.a. 151-End). The “nuclear distribution” gene *NudC* was first found in *A. nidulans*, in which *NudC* mutants prevented nuclear migration in the germ tubes of a filamentous fungus [37]. *NudC* genes are highly conserved among species from *Schizosaccharomyces pombe* to *Homo sapiens* [38], and contain a CS (CHORD-containing proteins and SGT1) domain [31,39]. The CS, HSP20-like, and p23-like domains belong to the HSP20 domain superfamily. The HSP20 domain superfamily has a CS domain, which is a β-sandwich fold consisting of eight strands in two β-sheets in a Greek-key topology [40], and its members are independent of or cochaperones of heat shock protein 90 (Hsp90) [39,41]. This evolutionally conserved sequence among phylogenetically diverse organisms may be linked to a conserved migration-associated function since the expressions of full-length human or rat NudC proteins can rescue nuclear migration defects in *Aspergillus* NudC mutants [42,43]. However, the detailed mechanism responsible for the effects of NudC and of its CS domain in nuclear migration is not known. In the present study, we found NAGK–NudC interactions in three different species, that is, in man (embryonic kidney cell line), in rats (migratory neurons generated from E14 rat cortex), and in mice (cortical sections of E17.5 mouse embryo), which also suggests that the role of the NAGK–NudC interaction in migration is evolutionally conserved. 

We also present biological evidence regarding the colocalization of and the interaction between NAGK and the NudC–Lis1–dynein complex. First, we found that NAGK colocalized with NudC at the leading projections of cultured migratory neurons (HEK293T cells) and in NPCs of the developing mouse cortex. A migratory phase of a neuron is characterized by a rostral position of the centrosome with respect to the nucleus in the migratory direction. In such migratory cells, we found MTs formed perinuclear cage-like structure at the rear and projecting into leading processes during migration, presumably to translocate nuclear MT structures to leading processes. The migratory phase was confirmed by DCX expression (a marker for migratory neurons) [44], and notably, NAGK colocalized with DCX in the leading processes of these neurons, which suggested its involvement in migration. Second, using a combination of PLA and ICC, we visualized the tripartite interaction between NAGK, NudC, and dynein. PLA showed NAGK first interacted with NudC, and that NAGK–NudC colocalized with DYNLRB1 around the nuclei and cell cortices of migrating cells. The formation of the NAGK–NudC–dynein complex was further verified using a high-resolution confocal microscope equipped with a 3D image module. Aumais et al. (2001) also showed that mammalian NudC interacted with the dynein complex in a coimmunoprecipitation assay. Since NAGK interacts with dynein complex ([24,29] and the present study), we conclude that NAGK interacts with NudC in the dynein complex. Third, using a similar experimental design, we found the NAGK–NudC–Lis1 complex localized around nuclei and the leading poles of migratory cells. Furthermore, NAGK–NudC PLA dots colocalized with Lis1 ICC signals in the same subcellular regions of migratory HEK293T cells and neurons. Together, these findings indicate that NAGK–NudC–Lis1 interact in dynein complex.

Morris et al. [34] also reported that NudC interacts with Lis1, and Capello et al. [17] showed that NudC–Lis1 plays a crucial role in nuclear movement and cell migration. Lis1 is an essential protein for migration and its mutation causes classical lissencephaly, which is characterized by defects in brain development characterized by a smooth cerebral surface, cortical lamination defects, mental retardation, and seizures [45,46]. However, little is known of the mechanism whereby Lis1 regulates neuronal migration. Lis1 has been reported to interact with dynein [8,47], and to act as an anchor for dynein at the cortices of *Drosophila* oocytes [48]. In accordance with the results of the present study, NudC was previously shown to colocalize with Lis1 and dynein during neuroepithelium development [8,34,49]. Aumais et al. [32] proposed a model of cooperation between NudC, Lis1, and dynein in the context of dynein function in migratory neurons, whereby NudC and Lis1 were involved in targeting and regulating dynein at cell cortices or interstitial junctions on substrates or at the leading pole of migrating neurons. However, NudC was reported not to interact directly with Lis1 or the Lis1/Ndel1 complex [39], which suggests that an as-yet unidentified linker is present in the dynein complex. Since the present study shows that NAGK interacts with NudC and Lis1 in the dynein complex, we propose that NAGK provides a link between NudC and Lis1 and promotes dynein activity. We previously reported that NAGK works as a bridging molecule in the dynein complex at Golgi outposts [25,29] and interacts with dynein during prophase nuclear envelope breakdown and metaphase chromosome separation [24]. Therefore, we suggest NAGK might also act as a modulator of dynein motor at the nuclear envelope and leading protrusions to mediate N-C coupling during cell migration.

The present study shows the functional significance of the interaction between NAGK with NudC and Lis1 in the dynein complex during cell migration. To verify these effects, we employed four different experimental migration models. First, we performed wound-healing assays [50], which showed that the exogenous overexpression of NAGK accelerated HEK293T cell migration, whereas shRNA-induced NAGK knockdown slowed migration. These phenomena are similar to the previously reported effects of NudC [36] or DYNLRB1 [51] knockdown or Lis1 inhibition effect on cell migration [36,52], and they provide evidence for the involvement of NAGK in cell migration. Second, we performed in vitro migration assays using neurospheres, which allowed NPCs to migrate outward on top of each other, which is believed to occur in vivo and is termed chain migration [53]. We found that neurons overexpressing exogenous NAGK migrated faster and that neurons transfected with NAGK shRNA migrated much slower than control neurons. Together with the finding that Lis1 depletion decreased neuronal migration in a similar assay [54], our findings support the role of NAGK, in association with Lis1, in cell migration. Third, we confirmed the role of NAGK in neuronal migration using an in utero electroporation study. NAGK knockdown significantly retarded neuron migration from VZ to CP in developing mouse embryonic brains, whereas NAGK overexpression promoted in vivo neuronal migration. Finally, we found that a peptide derived from the large domain of NAGK predicted by in silico molecular docking analysis to interact with NudC inhibited cell migration. This peptide significantly retarded the migrations of naive HEK293T and SH-SY5Y cells and of NAGK overexpressing HEK293T cells, thus further confirming the effect of NAGK–NudC interaction on cell migration. Together, these findings provide strong evidence of an interaction between NAGK with NudC and Lis1 in dynein complex, which, in turn, regulates neuronal and non-neuronal cell migration and brain development.

**Figure 8:**
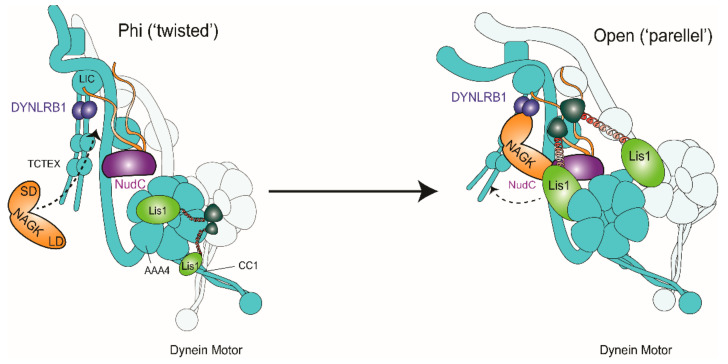
A proposed model for dynein activation by NAGK–NudC–Lis1 during cell migration. Lis1 plays a critical role in cell migration. Two β-propellers from the same Lis1 dimer bind to dynein: one to the ring (AAA4) (SiteRing) and the other to the stalk (coiled-coil 1; CC1) (SiteStalk) to induce low microtubule (MT) affinity for dynein, whereas in the high-MT-affinity state, a single β-propeller binds at SiteRing and keeps the motor tightly bound to the MT in preparation for cargo loading. DeSantis et al. (2017) [55]. Interaction of the small domain (SD) of NAGK with DYNLRB1 is supposed to “push up” the dynein light chain tailing, which would induce conformational changes of the dynein complex. This conformational change, together with the intervention of NudC and the large domain (LD) of NAGK, would trigger the release of one β-propeller from the dynein stalk and transform the dynein complex from the low MT-affinity phi state to the high-MT affinity open state in preparation for dynactin to cargo binding. Based on our finding that Lis1–NudC–NAGK–DYNLRB1 complexes are located near nuclei and cell cortices of leading edges of the migratory process, we propose that a “bivalent” NAGK binds to both DYNLRB1 and NudC and that the NAGK–NudC complex further interacts with the “free” β-propeller of the Lis1 dimer. TCTEX, Dynein Light Chain Tctex-Type.

Then, how does NAGK regulate dynein motor during cell migration? Dynein plays a central role in cell migration by pulling centrosomes and nuclei toward the (-) end of MT. Pulling a large cargo such as a nucleus at the molecular level probably requires the coordination of multiple dynein complexes. To date, Lis1 is the only dynein regulator known to interact directly with its motor domain. Lis1 may regulate dynein in two ways. First, at the motor and MT-binding side of the dynein complex, Lis1 may play a role in the loading of large cargoes, as multiple dyneins must be bound tightly to MT (+) ends. Lis1 has two quite different methods of regulating dynein because it can induce low or high affinity for MT [55]. These different regulatory modes depend on the stoichiometry of Lis1 to dynein binding. In the low-affinity state of “stepping” dynein, two β-propellers from one Lis1 dimer bind to dynein, that is, one to the ring (AAA4) (Site_Ring_) and the other to dynein’s stalk, more specifically coiled-coil 1 (CC1) (Site_Stalk_), the latter of which is required for the Lis1-induced weak MT-binding state. DeSantis et al. (2017) proposed that a single β-propeller binds at Site_Ring_ and maintains dynein in a high-MT-affinity state by keeping the motor tightly bound to MT in preparation for cargo loading [55]. This condition would be advantageous in terms of the work required to load a massive cargo such as a nucleus. In the present study, we found the Lis1–NudC–NAGK–DYNLRB1 complex near nuclei and cell cortices during the migratory phase. To explain the functional link between NAGK and this complex, we hypothesize that a “bivalent” NAGK binds to DYNLRB1 and NudC, and the NAGK–NudC complex interacts with the “free” β-propeller of Lis1 to link dynein to a nucleus or cell cortex during migration (Figure 8). 

Second, on the tail side of the dynein heavy chain, NAGK would help transform the dynein complex to prepare it for cargo loading. The activation of dynein is accompanied by a structural change from the phi to the open form and light chain (LC)—heavy chain (HC) tail separation [56]. The phi- to open-dynein transition also involves a change from a “twisted” to a “parallel” state of the 200-amino-acid N-terminal dimerization domain (NDD) of the HC tail. Interestingly, this latter transition is correlated with small shifts in the relative positions of intermediate chains (ICs) and larger changes in the positions of LCs: DYNLRB1 rotates through 25°, and LC8/Tctex density moves away from the HC neck [57]. Previously, we showed that the small domain of NAGK interacts with DYNLRB1 and NAGK plays a non-enzymatic structural role in dynein activation [25,26,27,29]. We consider that NAGK binding to DYNLRB1 causes a shift in the relative positions of ICs. More specifically, DYNLRB1 interacts with dynein intermediate chain (IC) through residues 79–82, 88, and 90 [58]. Interestingly, this DYNLRB1 interaction domain is also the NAGK binding site, as indicated in our previous report [29]. Furthermore, our molecular modeling and cell biological studies showed that residues 59 to 69 in the small domain of NAGK are major hotspots for the NAGK–DYNLRB1 interaction [28]. Therefore, there seems to be binding competition between NAGK and IC for DYNLRB1. If NAGK wins, NAGK may push DYNLRB1 to rotate through 25° and induce “twisted” to “parallel” transformation of NDD and “push up” the LCs tailing (Figure 8). Cryo-electron microscopy (Cryo-EM) studies are required to confirm this supposition.

## 4. Materials and Methods

### 4.1. Antibodies and Plasmids

The plasmids, including pDsRed2 vector, NAGK short hairpin (sh) RNAs and pDsRed2–NAGK, were used for transfection, as described previously [26,27]. Antibodies including NAGK mouse monoclonal (1:10 (PLA); Santa Cruz Biotechnology, Dallas, TX, USA)); NAGK chicken polyclonal (ICC (1:1000); GW22347, Sigma, St. Louis, MO, USA)); DYNLRB1/LC7 rabbit polyclonal (ICC (1:50) and PLA (1:25); Proteintech Group, Chicago, IL, USA)); Lis1 rabbit polyclonal (Santa Cruz); NudC rabbit polyclonal (ICC (1:50), Proteintech Group, Rosemont, IL)); doublecortin rabbit polyclonal (1:50; Genetex, Irvine, CA, USA); and alpha-tubulin mouse monoclonal (1:10; Developmental Studies Hybridoma Bank, University of Iowa, Iowa City, IA, USA). In order to detect primary antibodies, secondary antibodies with fluorescent level were used, including Alexa Fluor (488 and 568, Invitrogen, Carlsbad, CA, USA)

### 4.2. Experimental Animals

The Institutional Animal Care and Use Committee of the College of Medicine, Dongguk University, approved all animal experiments (Approval no: IUCAC-2018-06, March 03, 2018) beforehand [28]. On the 13th day of pregnancy, time-pregnant rats (Sprague–Dawley) were ordered and housed in a pathogenic free standard laboratory condition (18–25 °C, 45–65% humidity, light/dark cycle of 12/12 h) and maintained with free access to food and water ad libitum. All experiments were conducted by following the appropriate guidelines and regulations.

### 4.3. Protein-Protein Docking, Structural Modeling, and Peptide Design

The protein data bank (PDB) server was used for retrieving NudC (PDB id: 3QOR) and NAGK (PDB id: 2CH6) three-dimensional structures. The structures were fixed by adding hydrogen and correcting pH. The force field, Optimized Potential for Liquid Simulations (OPLS), was applied to minimize the energy of the structures [59,60], using Schrödinger 2017-1 (Schrödinger, LLC, New York, NY, USA, 2017). Protein–protein docking simulation was performed to predict the NAGK–NudC interaction in the SwarmDock server [61]. SwarmDock uses an algorithm that maintains the flexibility to predict protein–protein physical interaction [62]. After that, the FoldX [63] program was used to predict the binding energy of the docked complex. This program calculates ΔG in Kcal/mol for protein–protein complexes using an empirical force field algorithm, which considers electrostatic, van der Waals, H-bond, solvation, and entropic energies of protein–protein complexes [64].

Molecular dynamics simulation was conducted via YASARA dynamic software (*YASARA Biosciences* GmBH, Vienna, Austria) [65], and the lowest binding energy complex was used for molecular dynamics simulation, using the AMBER14 force field [65,66,67], as previously described [28,68,69]. We used the TIP3P solvation system (density of 0.997 g/L) to simulate the complex with a constant pH of 7.4, where the system neutralization was done by adding Na^+^ and Cl^−^ ions [70]. Energy minimization was applied to the system with a simulated annealing protocol, which uses the steepest descent approach. Electrostatic interactions that are in the long-range were described by the Ewald Particle Mesh (PME) method. By following Berendsen thermostat, the simulation was conducted by using multiple time-step algorithms [71,72] and performed for 100 ns with constant pressure. With an interval of 10 ps, trajectories were obtained in the simulation and analyzed through VMD [73] and YASARA tools. 

The NAGK peptides (Y^157^WIAHQAVKIVFDSI^171^ and L^198^GILTHLYRDF^208^) and NudC complexes were constructed from the lowest energy structures by free energy landscape analysis using Maestro Version 11.1.012. Peptide–protein complexes were also subjected to an additional 55 ns simulation using YASARA dynamic software following similar protocols. To select the peptide with better binding affinity, binding energy calculation was performed using the built-in MM-PBSA calculation. The mathematical details described previously [74,75].

### 4.4. Cell Culture, Transfection and Ni-NTA-Based Pull-Down Assay

HEK293T and SH-SY5Y cells were grown on coverslips (coated by polylysine) in Dulbecco’s Modified Eagle Medium (DMEM) (Invitrogen) supplemented with Penicillin Streptokinase (1%) and fetal bovine serum (10%). Lipofectamine^®^ 2000 reagent (Invitrogen) was used for cells transfection with indicated plasmids by following the manufacturer’s guidelines.

For pull-down assay, His-tagged NudC plasmid (pENTER-CMV-NudC, Vigene Biosciences, Rockville, MD, USA) was transiently transfected in HEK293T cells. The cells were lysed and collected after 48 h of incubation by using lysis buffer (25 mM Tris, 150 mM NaCl, 1.0 mM ethylenediamine tetraacetic acid (EDTA), 1% NP-40, 5% glycerol; pH 7.4) containing protease inhibitor cocktail (Thermo Scientific, Rockford, IL, USA), followed by washing with cold PBS. MagListo™ Ni-NTA magnetic silica resin (Bioneer, Daejeon, Korea) was allowed to bind with supernatant, which was then pulled down by following manufacturer’s instructions, as described earlier [28].

The interested proteins were detected from the elution by using immunoblotting with respective antibodies, anti-NAGK (1:1000; mouse monoclonal, Santa Cruz Biotechnology, TX, USA), anti-DYNC1LI1 (1:1000; mouse monoclonal, Millipore Sigma, Burlington, MA, USA), or anti-DYNLRB1 (1:1000, rabbit polyclonal, ABclonal, MA, USA), anti-Lis1 (1:500, rabbit polyclonal, Santa Cruz; Dallas, TX, USA) followed by 15% Tricine-SDS-PAGE separation and polyvinylidene difluoride (PVDF) membrane blotting. The blots were detected using an enhanced chemiluminescence (ECL) detection kit (Invitrogen, Waltham, MA, USA). To strip membranes, stripping buffer was used (Pierce Biotechnology, Rockford, IL, USA), and hybridization was performed with different primary and secondary antibodies.

### 4.5. Wound-Healing and Peptide Inhibition Assays

A wound-healing assay was conducted on 80% confluent cells, grown in 12-mm diameter coverslip (coated with polylysine), as mentioned earlier [50]. A sterile pipette tip was used to scratch cell monolayers, and then debris was cleaned by washing and maintained in fresh medium. For ICC and PLA, migrating cells were fixed 48 h post-scratching and antigen–antibody reactions were performed. To investigate overexpression and knockdown effects, indicated plasmids were transfected initially, scratched, and then photographed at indicated times under a fluorescent microscope. 

For peptide inhibition assays, cells were transfected by lipofectamine^®^ 2000 with pDsRed2 or pDsRed2–NAGK plasmids at 50% of cell confluency. Then, transfected cells are incubated for 48 h until confluent. Then, a linear scratch was made by using a pipette tip, and cells were washed and transfected with 1 μg Y^157^WIAHQAVKIVFDSI^171^ peptide (GL Biochem Ltd.; Shanghai, China) via Pierce protein transfection reagent (ThemoFisher, Waltham, MA, USA), as mentioned previously [28]. 

Migration activity was quantified as the percentage of migrating cells moving with the migratory front. When peptide was added directly to the medium, cells were incubated in a serum-free medium for 4 h. A linear scratch was made by using a pipette tip and washed. Cells were incubated with either peptide (25 μM) or DMSO containing low serum (1% FBS) medium. Cell migration rates were quantified by expressing recovered areas as percentages to total damaged areas.

### 4.6. Culture of E14 Neuronal Progenitor Cells (NPCs) and Neuros–Phere Migration Assays

NPCs were cultured, as mentioned earlier [76]. In brief, embryos (E14) were isolated from a Sprague–Dawley rat. Cortices were aseptically dissected from brains and dissociated to produce single-cell suspensions by gentle mechanical pipetting using fire-polished Pasteur pipettes. In the presence of epidermal growth factor (10 ng/mL) and fibroblast growth factor-2 (10 ng/mL), cells were then grown overnight in 5 mL of medium supplemented with DMEM-F12/Glutamax (Gibco, ThemoFisher, Waltham, MA, USA), penicillin–streptomycin (1%, Gibco) and B27 supplement (1%, Gibco) in 25 cm^2^ flasks [77]. For ICC and PLA, coated coverslips (poly-D-lysine/laminin) were used to plate floating neurospheres. The transfection of neurospheres with specified plasmids was done by Lipofectamine^®^ 2000 reagent (Invitrogen). Geltrex^®^ matrix (Gibco) was used for transfection and live-cell imaging. The plated neurospheres were further grown for 48 h to attain differentiated cortical neurons in the medium containing 2% B27 supplement, 500 mM L-glutamine, and 1% penicillin–streptomycin with no growth factors. After culturing for 48–60 h, differentiated cortical neurons cultures were used for live-cell imaging and ICC.

### 4.7. In Utero Electroporation (IUE) and Immunohistochemistry

In utero electroporation (IUE) was conducted following the previously described method [78]. After anesthetizing by isoflurane, the embryos in the uterus of pregnant mice were microinjected with plasmids by using pulled glass capillaries. The injection was done in the lateral ventricles of embryos in the uterus. Before injections, laparotomy was used to expose uterine horns. First, 1.0 μL of plasmid solution containing fast green (0.01%, Sigma-Aldrich, St. Louis, MO, USA) was injected with indicated plasmids (0.5 μg) to the embryos. A square-wave pulse generator (ECM 830; BTX, Holliston, MA, USA) was used to perform electroporation by discharging five pulses (50 ms long, 45 V) with 950 ms intervals [79] using Tweezertrodes (BTX, USA) with a diameter of 5 mm. Then, by returning uterine horns to the abdominal cavity, the electroporated embryos were grown naturally until it is required. After that, a fixation protocol was applied to fix electroporated embryonic brains, which follows 2 h fixation by 4% paraformaldehyde at 4 °C and 16 h incubations in 30% sucrose containing PBS at 4 °C. Tissue-Tek OCT (Sakura Finetek, USA) was used to embed brains for cryosectioning at 10 μm. Tissue sections were transferred into coverslips coated with poly-lysine, which were then washed PBS blocking solution containing 0.05% Triton X-100 and 5% normal goat serum. For immunohistochemistry, incubation of the primary antibody, as indicated previously, was done overnight at 4 °C, which then follows incubation for 90 min at room temperature with specific secondary antibodies with fluorophore conjugation. After washing, specimens were mounted using mounting medium onto glass slides. 

### 4.8. Immunocytochemistry (ICC) and Proximity Ligation Assay (PLA)

Cells were fixed by sequential fixed in paraformaldehyde/methanol, and ICC was carried out as previously described [80]. According to the manufacturer’s instructions, in situ PLA was done by using a Duolink assay kit (Olink Bioscience, Uppsala, Sweden) with slight modifications [29]. For the combination assay of ICC and PLA, we first performed the PLA experiment and then followed the ICC procedure.

### 4.9. Image Acquisition

An Olympus microscope BX53 was used to take the fluorescence images containing DAPI staining. A Leica research microscope (DM IRE2) (Leica Microsystems AG, Wetzlar, Germany) was used for taking fluorescence images in Figure 5 and Figure 6. Figure 3E,F were made by confocal microscopy (Leica TCS SP8 microscope and LAS X software, Leica Microsystems, ver. 2.0.2.15022) [81], using the facilities of the Advanced Neural Imaging Center of the Korean Brain Research Institute (KBRI). We used Adobe Systems Photoshop 7.0 software (Adobe, San Jose, CA, USA) for processing digital images.

### 4.10. Statistics 

Three different experiments were considered for wound-healing assays. For counting, cells not more than 500 were considered from the various positions of each coverslip after transfecting for 48 h and 72 h, respectively. For in vitro migration assays, distances migrated by neurons (*n* = 20) in three different experiments were measured. For the in utero electroporation study, numbers of transfected neurons in cortical plates and intermediate and ventricular zones were calculated and described as total neurons percentages. Counting was done in a blinded way, which was carried out by independent experienced investigators. Within each cohort, the animal selection was made from random cages at a random time and season. By using natural intelligence, the sample size was chosen depending on the data variability; however, not less than *n* = 5. In order to compare two groups, the Student’s t-test was used, while multi-group comparisons were made by one-way ANOVA with Duncan’s multiple comparison post hoc test. The use of parametric tests was verified by the normality test. The predictions were considered statistically significant when the *p* values were <0.05. *p* values of <0.0001 indicated a highly significant prediction. Statiscal Software, GraphPad Prism v 8.0 (GraphPad Software, San Diego, CA, USA) and SPSS 19.0 (SPSS Inc., Chicago, IL, USA) were used for statistical calculations, where the data are presented as means ± SDs.

## 5. Conclusions

In summary, the present study shows NAGK interacts with NudC and Lis1 in the dynein complex and thus promotes cell migration. Our data suggest the possibility that NAGK recruits NudC and Lis1 to make a NudC–NAGK–Lis1 tripartite interaction in the dynein complex. Based on these findings, we propose a dynein activation model involving “bivalent” NAGK, where the small domain of NAGK interacts with DYNRB1 to transform the NDD of dynein HC from the phi to the open state, “pushing up” the LCs tailing. Alternatively, the large domain of NAGK recruits NudC and binds Lis1 to coordinate motor power at high loads, such as pulling a nucleus in N-C coupling during migration.

## Data Availability

All data generated or analyzed during this study are included in this published article (and its Supplementary Information Files).

## Supplementary Materials

The following are available online at https://www.mdpi.com/1422-0067/22/1/129/s1, Figure S1: In silico protein-protein docking, molecular dynamics simulation and peptide binding energy evolution, Figure S2: Overexpression of NAGK promoted and NAGK shRNA transfection decreased cell migration.

## Abbreviations

AMBER14	Assisted Model Building with Energy Refinement Version 14
CP	Cortical Plate
Cryo-EM	Cryogenic Electron Microscopy
DAPI	4′,6-diamidino-2-phenylindole
DCX	Doublecortin
DMEM	Dulbecco’s Modified Eagle Medium
DMSO	Dimethyl Sulfoxide
DYNLRB1	Dynein Light Chain Roadblock Type 1
EDTA	Ethylenediamine Tetraacetic Acid
ECL	Enhanced Chemiluminescence
HC	Heavy Chain
ICC	Immunocytochemistry
IUE	In Utero Electroporation
IR	Immunoreactive
Izs	Intermediate Zones
LC	Light Chain
LD	Large Domain
Lis1	Lissencephaly 1
MM-PBSA	Molecular Mechanics Poisson–Boltzmann Surface Area
MT	Microtubule
NAGK	N-acetylglucosamine Kinase
N-C	Nucleus–Centrosome
NDD	N-Terminal Dimerization Domain
Ni-NTA	Nickel NTA
NPCs	Neuronal Progenitor Cells
NudC	Nuclear Distribution Protein C
NudE	Nuclear Distribution Gene E Homolog 1
OPLS	Optimized Potential For Liquid Simulations
PBS	Phosphate Buffered Saline
PVDF	Polyvinylidene Difluoride
PDB	Protein Data Bank
PLA	Proximity Ligation Assay
PME	Particle Mesh Ewald
RFP	Red Fluorescent Protein
SD	Small Domain
RMSD	Root Mean Square Deviation
RMSF	Root Mean Square Fluctuation
RNAi	RNA Interference
shRNA	Short-Hairpin RNA
SVZ	Subventricular Zone
TIP3P	Transferable Intermolecular Potential Three Points
UDP–GlcNAc	Uridine Diphosphate N-acetylglucosamine
VMD	Visual Molecular Dynamics
VZs	Ventricular Zones
