# Supplementary material for: N-Acetyl-d-Glucosamine Kinase Interacts with NudC and Lis1 in Dynein Motor Complex and Promotes Cell Migration
Source: Int J Mol Sci. 2020 Dec 24;22(1):129. doi: 10.3390/ijms22010129 (PMC7795690; doi:10.3390/ijms22010129)
Supplement: Supplementary file 1 [file ijms-22-00129-s001.pdf]

Supplementary File

A

Complex Name	Docking Score	Binding Energy
NAGK-NudC	-29.35 kcal/mol	-8.79 kcal/mol

B

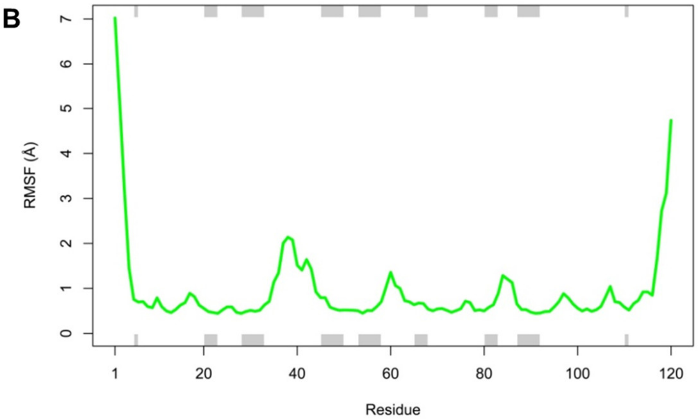

Figure S1: ***In silico*** **protein-protein docking, molecular dynamics simulation and peptide binding energy evolution.** A. Protein-Protein docking score and binding energy evolution of NAGK-NudC complex. B. Protein thermodynamic stability during the molecular dynamics simulation was evaluated using root-mean-square fluctuations (RMSFs) for NudC by considering protein backbone atoms (C, Cα, and N).

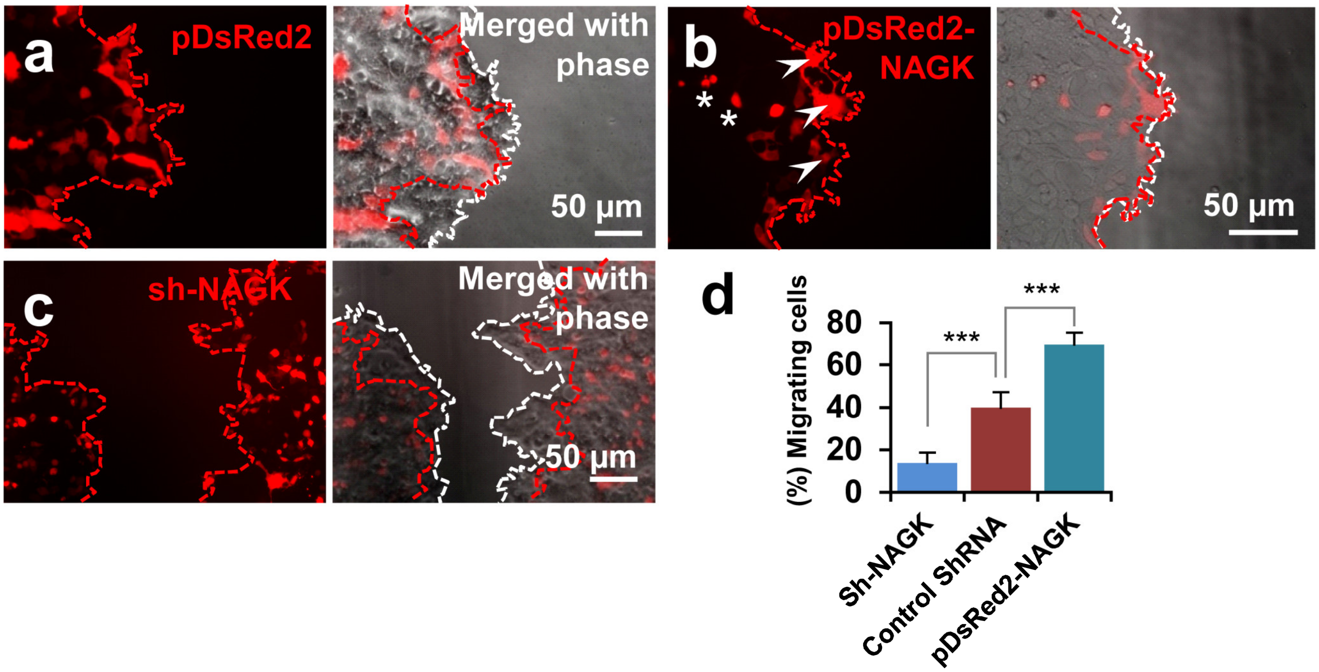

Figure S2: Overexpression of NAGK promoted and NAGK shRNA transfection decreased cell migration. A. Wound-healing assay. HEK293T cells were transfected with indicated plasmids, and the wound-healing assay was performed as described in Materials and Methods. Epifluorescent live-cell images showing cells transfected with the indicated plasmids merged with phase-contrast images. The white dotted line shows the leading edge of migratory cells and red dotted line shows the movement of transfected cells. Cells transfected with a control plasmid (pDsRed2) were distributed evenly throughout migratory and non-migratory areas (a). In contrast, cells transfected with pDsRed2-NAGK were mostly positioned at the migration front (b), whereas cells co-transfected with pDsRed2 vector and NAGK shRNA (sh-NAGK) were positioned in non-migratory areas (c). Scale bar; 50 μm. Transfected cells present at the migratory front were considered migrating cells and their numbers are plotted on a bar diagram as percentages of all transfected cells in migratory areas (d). Cells treated with exogenous NAGK (pDsRed2-NAGK) were present at significantly higher percentages at migratory fronts, and percentages of cells treated with NAGK shRNA (sh-NAGK) were significantly lower than those of pDsRed2 transfected controls. ***, $p < 0.01$, n=500. B.
